# High-resolution view of compound promiscuity

**DOI:** 10.12688/f1000research.2-144.v2

**Published:** 2013-07-26

**Authors:** Ye Hu, Jürgen Bajorath

**Affiliations:** 1 Department of Life Science Informatics, B-IT, LIMES Program Unit Chemical Biology and Medicinal Chemistry, Rheinische Friedrich-Wilhelms-Universität, Bonn, Germany

## Abstract

Compound promiscuity is defined as the ability of a small molecule to specifically interact with multiple biological targets. So-defined promiscuity is relevant for drug discovery because it provides the molecular basis of polypharmacology, which is increasingly implicated in the therapeutic efficacy of drugs. Recent studies have analyzed different aspects of compound promiscuity on the basis of currently available activity data. In this commentary, we present take-home messages from these studies augmented with new results to generate a detailed picture of compound promiscuity that might serve as a reference for further discussions and research activities.

## Introduction

Polypharmacology is an emerging theme in drug discovery ^1, 2^. It is generally accepted that drugs often elicit their therapeutic effects through interactions with different targets and the ensuing modulation of multiple signaling pathways. In some therapeutic areas such as oncology, polypharmacology is heavily exploited, for example, through the use of promiscuous ATP site-directed protein kinase inhibitors ^3^. In other areas, such as the treatment of infectious or chronic inflammatory diseases, achieving a high degree of target selectivity of drug candidates plays a major role.

The study of drug polypharmacology has become an important topic in pharmaceutical research ^4, 5^, especially focusing on combined computational and experimental analysis ^5^. On the basis of drug-target networks, it was estimated early on that a drug interacts on average with approximately two targets ^4^. More recent estimates from computational data analysis suggest that drugs might bind on average to two to seven targets, depending on the primary target families, and that more than 50% of current drugs might interact with more than five targets ^6^.

Compound promiscuity as defined herein is the origin of polypharmacology. Promiscuity analysis can be extended from drugs to bioactive compounds through computational mining of currently available activity data. The results of activity data analysis are generally affected by data incompleteness ^7^. This potential influence can only be eliminated by reaching the ultimate (and probably elusive) goal of chemogenomics ^8^, i.e., testing all compounds against all targets. In the presence of data incompleteness, compound promiscuity rates are likely underestimated. However, it is not certain that further increasing amounts of assay data will indeed significantly alter the currently emerging view of compound promiscuity ( *vide infra*).

Recent studies have generated a differentiated picture of compound promiscuity. The interested reader is also referred to comprehensive reviews of compound promiscuity analysis ^9^ and polypharmacology ^6^. In this commentary, we summarize key messages from recent promiscuity analysis in a compact format. It is hoped that this summary might be helpful as a reference for further studies.

## Key results of compound promiscuity analysis

Public data sources for compound promiscuity analysis discussed herein have been ChEMBL ^10^, the major repository of compound activity data from medicinal chemistry (currently in May 2013 containing 1,295,510 compounds with a total of 11,420,351 activity annotations), the PubChem BioAssay database ^11^, the major repository of screening data (with more than 3300 confirmatory assays), and DrugBank ^12^, which currently contains 1518 approved and 5080 experimental drugs.

It is important to note that collecting all activity annotations for a compound reported in the literature including, for example, reporter gene or other cell-based assays is at best providing a measure of assay promiscuity, but not of specific interactions with different targets ^9^. Therefore, it is generally required to apply data confidence criteria such as the presence of well-defined activity measurements or evidence for direct ligand-target interactions ^9^ (as provided in ChEMBL as activity data filters).

### Activity measurement dependence

When monitoring the growth of compound activity data in ChEMBL over a period of more than two years from its original release (January 2010) to release 13 (May 2012), a significant increase in the number of promiscuous compounds was detected ^13^. However, by quantifying compound-based target relationships, it was determined that the *increase in compounds with activity against targets from different families was largely due to (assay-dependent) IC _50_ measurements, rather than (assay-independent) equilibrium constants (K _i_ values)* ^13^. IC _50_ values are easier to determine than K _i_ values and provide the readout of most primary biochemical assays (except single-point screening assays), which might at least in part rationalize greater target coverage and the IC _50_-dependent increase in compound promiscuity across different families. However, it can also not be excluded that apparent promiscuity in different assays is higher on the basis of IC _50_ measurements, given their assay dependence (and often limited accuracy). Regardless, the type of activity measurements that are taken into account influences the outcome of promiscuity analysis. Thus, clear specification of activity measurements and data selection criteria are required.

The subset of compounds with available K _i_ measurements from ChEMBL release 13 was further investigated. *On the basis of K _i_ measurements, approximately 62% of all compounds were only annotated with a single target, ~36% with two or more targets from the same family, and only ~2% of all active compounds with multiple targets from different families* ^14^. *A promiscuous bioactive compound was found to interact on average with two to three targets*.

Accordingly, compounds that display intra-family promiscuity might also be considered as candidates for privileged structures/compounds that are preferentially active against targets from a particular family. Therefore, these compounds can be distinguished from those that are promiscuous across different target families.

### Activity data from different sources

One might anticipate that the degree of compound promiscuity would be particularly high in screening assays (even if frequent hitters and other non-specific compounds are excluded). Therefore, 1085 confirmatory bioassays from PubChem were systematically analyzed. It was found that ~77% of all confirmed active compounds were tested in more than 50 different assays ^15^. Thus, these active PubChem compounds provided a sound basis for promiscuity assessment. These results were in part surprising. *An active PubChem compound displayed a ~50% probability to interact with two or more targets. The probability to interact with more than five targets was only ~8%. On average, a PubChem screening hit was active against 2.5 targets*. For comparison, *compounds from the IC _50_- and K _i_-based subsets of ChEMBL release 14 (August 2012) interacted on average with 1.4 and 1.7 targets, respectively* ^15^. The comparably low ratios observed for both compound subsets indicated that IC _50_ measurements did not systematically increase promiscuity rates ( *vide supra*). The analysis of active compounds from PubChem confirmatory assays provided an upper level estimate of promiscuity, which was not significantly higher than that for ChEMBL compounds.

### Prevalent promiscuity profile

Detailed analysis of compound activity data from ChEMBL release 14 (August 2012) has made it possible to derive a *promiscuity profile* that is most characteristic of bioactive compounds from medicinal chemistry sources. *The majority of currently available promiscuous compounds is active in the sub-µM range against two to five targets from the same family and displays potency differences against these targets within one or two orders of magnitude* ^16^. An important aspect of this representative profile is that promiscuity does not imply low potency. Furthermore, compounds that are highly potent against a (primary) target and weakly potent against others are not frequently found ^16^.

## Up-to-date promiscuity rates

In Table 1, current average promiscuity rates are summarized for compounds from ChEMBL, PubChem, and DrugBank. For promiscuity assessment of drugs, all targets reported in DrugBank were considered.

**Table 1.** Average promiscuity of different compound categories.

Compound categories		Avg. # targets/compound
**ChEMBL 14/all bioactive compounds**	**K _i_**	1.7
	**IC _50_**	1.4
**DrugBank/drugs**	**Approved**	5.9
	**Experimental**	1.8
**PubChem/active compounds**		2.5
***ChEMBL 14/promiscuous compounds***	***K _i_***	*2.9*
	***IC _50_***	*2.7*
***DrugBank/promiscuous drugs***	***Approved***	*6.9*
	***Experimental***	*4.7*
***PubChem/promiscuous active compounds***		*3.7*

The average number of targets is reported for compounds from ChEMBL release 14 (divided into K _i_ and IC _50_ value-based subsets), approved or experimental drugs from DrugBank 3.0, and active compounds from PubChem confirmatory bioassays. Corresponding statistics are provided in italics for promiscuous compounds (having two or more target annotations). For compounds from ChEMBL, only high-confidence activity annotations were taken into account (i.e., explicit activity measurements with the highest confidence level of direct ligand-target interactions). For calculations on drugs, all DrugBank target categories were taken into account.

If all compounds with single or multiple target annotations are analyzed, ChEMBL compounds interact on average with one to two targets and PubChem compounds with two to three. However, approved drugs have on average close to six targets. In contrast, the degree of promiscuity of experimental drugs is considerably lower, with less than two targets per drug candidate. If only promiscuous compounds or drugs are taken into account (i.e., if compounds with single target annotations are excluded), promiscuity rates only slightly increase by about one target per compound, the exception being experimental drugs whose average number of targets increases from 1.8 to 4.7. Furthermore, median promiscuity rates were also calculated for promiscuous compounds from different sources, i.e., ChEMBL compounds with activity against at least two targets (K _i_ and IC _50_), approved and experimental drugs annotated with more than four or at least two targets, respectively, and PubChem compounds active against at least three targets. Compared to the average promiscuity rates reported in Table 1, the median rates were consistently lower. However, the differences between the average and median rates were small, i.e., less than one for ChEMBL and PubChem compounds. By contrast, differences were larger than one for approved and experimental drugs, i.e., on the basis of median rates, drug target numbers were reduced by 1.9 and 2.7, respectively. Hence, average promiscuity rates for drugs were likely biased by highly promiscuous drugs.

In Table 2, the probability of promiscuity is reported for compounds from different sources (calculated from target distributions of compounds). For a ChEMBL compound with available IC _50_ and K _i_ measurements, the current probability of activity against two or more targets is ~25% and ~38%, respectively (if both IC _50_ and K _i_ measurements were available for a compound, they were separately considered). However, for activity against more than five targets, the probabilities are reduced to only ~1%. Similar observations are made for confirmed PubChem screening hits (providing an upper-limit promiscuity assessment for bioactive compounds, *vide supra*). In this case, the probability of activity against two or more, or against more than five targets is ~51% and ~8%, respectively. Furthermore, the probability of promiscuity of approved drugs from DrugBank is ~84% and the probability to interact with more than five targets still ~37%. For experimental drugs, the corresponding probabilities are much lower, with only ~24% and ~3%, respectively.

**Table 2.** Probability of promiscuity.

Compound categories		# Targets	Probability (%)
**ChEMBL 14/all bioactive compounds**	**K _i_**	≥ **2**	37.9
		**> 5**	1.2
	**IC _50_**	≥ **2**	24.7
		> **5**	0.8
**DrugBank/drugs**	**Approved**	≥ **2**	84.1
		> **5**	37.4
	**Experimental**	≥ **2**	23.6
		> **5**	3.4
**PubChem/active compounds**		≥ **2**	50.9
		> **5**	7.6

For different compound categories and activity measurements, the probability of a compound to be active against two or more targets or more than five targets is reported.

## Compound promiscuity for different target families

All available compounds active against targets belonging to the five target families, including G protein-coupled receptor (GPCR) class A, protein kinases, ion channels, proteases, and nuclear hormone receptors, were assembled from ChEMBL release 14 and separated into K _i_ and IC _50_ value-based subsets, as described above. Average promiscuity rates were calculated for all compounds active against a given family as well as compounds active against multiple targets within the family, as reported in Table 3. With the exception of the K _i_ subset of the ion channel family, promiscuity degrees for compounds active against these target families were similar to those reported in Table 1. In Table 4, the probability of promiscuity (i.e., activity against at least two or more than five targets) is reported for compounds active against these families (according to Table 2). Similar observations were made. A significant relative increase (~10%) in probability of promiscuity was only observed for compounds active against two or more targets from the nuclear receptor family on the basis of the IC _50_ subset. Thus, for prominent target families, no above-average compound promiscuity rates were detected.

**Table 3.** Average promiscuity of compounds active against prominent target families.

Family	K _i_			
	# Targets	# Compounds	Avg. # targets/compound	
			All	*Promiscuous*
**GPCR class A**	121	21,754	1.7	*2.8*
**Kinases**	74	1151	1.4	*2.4*
**Ion channels**	22	1086	1.2	*3.9*
**Proteases**	90	4488	1.5	*2.8*
**Nuclear receptors**	13	901	1.4	*2.6*
Family	IC _50_			
	# Targets	# Compounds	Avg. # targets/compound	
			All	*Promiscuous*
**GPCR class A**	135	16,968	1.3	*3.0*
**Kinases**	192	13,316	1.3	*2.7*
**Ion channels**	52	4150	1.1	*2.2*
**Proteases**	108	11,833	1.6	*3.0*
**Nuclear receptors**	26	3782	1.4	*2.1*

From ChEMBL release 14, K _i_ and IC _50_ value-based compound subsets active against targets belonging to five prominent families were collected. The average number of targets is reported for compounds from individual target families. In addition, corresponding statistics are provided (in italics) for promiscuous compounds only (i.e., compounds having two or more target annotations within the family).

**Table 4.** Probability of promiscuity for compounds active against prominent target families.

Family	Probability (%)			
	K _i_		IC _50_	
	≥ 2 Targets	> 5 Targets	≥ 2 Targets	> 5 Targets
**GPCR class A**	39.1	0.8	16.0	0.8
**Kinases**	26.3	0.4	20.1	1.0
**Ion channels**	8.4	0.1	5.3	0.02
**Proteases**	27.5	0.5	27.2	1.0
**Nuclear receptors**	24.9	1.2	34.8	0.03

For compounds active against different target families, the probability of promiscuity (activity against two or more targets and activity against more than five targets) is reported.

## Promiscuity of compounds with increasing molecular weight

The degree of promiscuity was also determined for compounds with different sizes, i.e., molecular weight (MW). Seven subsets of compounds with increasing MW were collected from ChEMBL release 14 and organized into K _i_ and IC _50_ value-based subsets, as reported in Table 5. Average promiscuity rates of compounds with increasing MW were found to be comparable to the global rates. However, a significant relative increase in promiscuity was observed for the smallest compounds with MW ≤ 200 in K _i_ subset. Furthermore, the probability of activity against two or more targets also increased by more than 10% for the smallest compounds in both subsets, as reported in Table 6. For larger compounds across all MW ranges, no significant increases in promiscuity were observed compared to the global degree and probability of compound promiscuity reported in Table 1 and Table 2, respectively.

**Table 5.** Average promiscuity of compounds with different molecular weight.

MW range	K _i_		
	#Compounds	Avg. # targets/compound	
		All	*Promiscuous*
**≤ 200**	786	2.7	*4.1*
**(200, 300)**	3949	2.0	*3.2*
**(300, 400)**	10,913	1.8	*2.9*
**(400, 500)**	11,501	1.6	*2.7*
**(500, 600)**	6015	1.6	*2.8*
**(600, 700)**	1878	1.6	*3.0*
**> 700**	1497	1.7	*2.8*
MW range	IC _50_		
	#Compounds	Avg. # targets/compound	
		All	*Promiscuous*
**≤ 200**	1022	1.6	*2.8*
**(200, 300)**	9627	1.6	*2.8*
**(300, 400)**	25,190	1.4	*2.7*
**(400, 500)**	26,358	1.4	*2.6*
**(500, 600)**	12,534	1.4	*2.6*
**(600, 700)**	3247	1.3	*2.8*
**> 700**	2495	1.4	*2.9*

From ChEMBL release 14, compounds were selected and divided into seven subsets with increasing MW. The average number of targets is reported for compounds in all MW ranges. In addition, corresponding statistics are provided (in italics) for promiscuous compounds only (i.e., compounds having two or more target annotations).

**Table 6.** Probability of promiscuity of compounds with different molecular weight.

MW range	Probability (%)			
	K _i_		IC _50_	
	≥ 2 Targets	> 5 Targets	≥ 2 Targets	> 5 Targets
**≤ 200**	53.8	9.9	36.0	2.4
**(200, 300)**	44.9	2.4	32.7	0.9
**(300, 400)**	39.4	1.2	26.0	0.9
**(400, 500)**	37.3	0.7	22.8	0.7
**(500, 600)**	31.5	0.6	22.1	0.7
**(600, 700)**	30.7	0.9	17.6	0.7
**> 700**	38.3	1.2	18.9	0.3

For compounds with different molecular weight, the probability of promiscuity (activity against two or more targets and activity against more than five targets) is reported.

## Conclusions

Herein, we have provided a detailed and up-to-date view of compound promiscuity, the molecular basis of polypharmacology. For active compounds from medicinal chemistry and biological screening sources, the degree of promiscuity is lower than for drugs. There is a notable increase in promiscuity from bioactive compounds over drug candidates to approved drugs. The exploration of possible reasons for this apparent "promiscuity enrichment" along the drug discovery pathway should provide interesting opportunities for future research. On the basis of currently available high-confidence activity data, promiscuity of bioactive compounds is limited (and very low across different target families). However, if compounds are promiscuous, they typically bind to their targets with relatively high potency. Given the overall low degree of promiscuity of bioactive compounds including screening hits in the presence of nearly exponential data growth in recent years, it remains an open question if future chemogenomics efforts might substantially change the current picture of compound promiscuity ( *vide supra*). The majority of available bioactive compounds have single target annotations and we believe it is unlikely that most of them will display a high degree of currently undiscovered promiscuity. Hence, we would also conclude that the target specificity paradigm that has long dominated small molecule discovery efforts should continue to play a major role, despite emerging "anti-reductionism" and the increasing focus on phenotypic readouts.
