# Supplementary material for: High-resolution view of compound promiscuity
Source: F1000Res. 2013 Jul 26;2:144. Originally published 2013 Jun 27. [Version 2] doi: 10.12688/f1000research.2-144.v2 (PMC3799544; doi:10.12688/f1000research.2-144.v2)
Supplement: Peer review history

# Peer review: Referee response for version 2

**Published:** 2013-08-01

**Authors:** Stefan Laufer

**Affiliations:** 1 University of Tübingen, Tübingen, Germany

as already said, a very valid approach for my (kinase) community to deal with multi-target compounds

I have read this submission. I believe that I have an appropriate level of expertise to confirm that it is of an acceptable scientific standard.

# Peer review: Referee response for version 2

**Published:** 2013-07-29

**Authors:** Jeremy Jenkins

**Affiliations:** 1 Centre for Protein Chemistry, Novartis Institutes for BioMedical Research, MA, USA

My questions from v1 were sufficiently answered.

I have read this submission. I believe that I have an appropriate level of expertise to confirm that it is of an acceptable scientific standard.

# Peer review: Referee response for version 1

**Published:** 2013-07-16

**Authors:** Jeremy Jenkins

**Affiliations:** 1 Centre for Protein Chemistry, Novartis Institutes for BioMedical Research, MA, USA

Hu and Bajorath’s update on compound promiscuity in public compound bioactivity databases is timely with the increasing cognizance of polypharmacology and its role in the efficacy and safety of drugs. The article aims to raise reader awareness and present new questions to be answered, and so the title, abstract, and content are appropriate. All data are freely available for download for primary sources mentioned.

The conclusions are fair and unbiased.

Additional questions do arise from this survey. First, does the average number of targets per compound differ from the median (or do highly promiscuous compounds skew the average?).

Second, is it reasonable to begin distinguishing promiscuous from privileged compounds? For example, by incorporating target class information, staurosporine might be viewed differently from quercetin, where the former represents a highly privileged scaffold among kinases and the latter displays IC50 values against an abundance of target types. 

Third, the drug discovery field needs to understand if the "promiscuity enrichment" that occurs between the screening hits phase to the marketed drug phase largely reflects the depth of bioactivity data coverage for drugs, as drugs are highly profiled globally. The hit rates of drugs and medchem compounds across the same set of assays and targets would be needed to definitively conclude that drugs are more promiscuous. 

However, the apparent increased promiscuity of drugs supports the growing resurgence of phenotypic screening, the impetus for exploring compound combinations in the context of multiple genotypes, and begs the question of how medchem optimization of multiple targets should be attacked.

I have read this submission. I believe that I have an appropriate level of expertise to confirm that it is of an acceptable scientific standard.

# Peer review: Referee response for version 1

**Published:** 2013-07-09

**Authors:** Hans Matter

**Affiliations:** 1 Sanofi-Aventis, Paris, France

This interesting manuscript presents a view on compound promiscuity based on in-vitro data and the number of potential targets per compound in public databases such as ChEMBL, PubChem and DrugBank. In particular the authors investigate and challenge the notion that most compounds today in lead findings are active on a large multitude of biological data. The title is appropriate for this contribution and the abstract sufficiently summarizes this study. The conclusions are balanced and justified on the basis of the data analysis; this is therefore an essential view on the number of targets.

It is an interesting observation from this study that DrugBank annotated drugs appear to interact with a higher number of molecular targets compared to early phase compounds in ChEMBL or PubChem. Any interpretation of this finding should be treated with caution, but it is tempting to discuss from a partially historical view as DrugBank may be enriched with older drugs that would have been subjected to less strict requirements for in-vitro selectivity than in today’s drug discovery. In addition during and after approval, drugs may have been tested in more profiling assays as is the case with earlier screening-type substances.

Following the authors, this interesting argument also supports target-specific drug discovery paradigms used in past years. However, working with public databases leads to many caveats, all of which have been pointed out earlier, e.g. incompleteness of the data matrix and differences of data from different sources. It might be interesting for future investigations to cross-check this conclusion for compounds targeting families like kinases or GPCRs. Due to the challenges of inherent selectivity in those families one could expect a larger percentage of promiscuous compounds. The same discrimination might possibly be true for smaller versus larger compounds.

I have read this submission. I believe that I have an appropriate level of expertise to confirm that it is of an acceptable scientific standard.

# Peer review: Referee response for version 1

**Published:** 2013-07-01

**Authors:** Stefan Laufer

**Affiliations:** 1 University of Tübingen, Tübingen, Germany

Excellent work. My field (“the kinase community”) will benefit a lot form this commentary as compound promiscuity is an issue.

I have read this submission. I believe that I have an appropriate level of expertise to confirm that it is of an acceptable scientific standard.
